# The potential link between inflammatory bowel disease and male erectile dysfunction: mechanistic insights and novel therapeutic perspectives

**DOI:** 10.3389/fimmu.2025.1701741

**Published:** 2026-01-05

**Authors:** Shuxin Li, Hongliang Cao, Yuwei Liang, Chengsen Lv, Yutao Ma, Tong Yang, Bo Yuan, Wei Wei

**Affiliations:** Department of Urology, The First Hospital of Jilin University, Changchun, China

**Keywords:** inflammatory bowel disease, Crohn’s disease, ulcerative colitis, erectile dysfunction, inflammatory factors, therapy

## Abstract

This review provides a comprehensive synthesis of the link between inflammatory bowel disease (IBD) and male erectile dysfunction (ED), with a distinct emphasis on underlying mechanisms and novel perspectives. We critically evaluate the evidence and then systematically elucidate the “gut-penis axis” detailing how gut-derived signals orchestrate a systemic inflammatory response that culminates in penile vascular dysfunction. A novel aspect of our work is the integration of psychological factors into a cohesive psychoneuroimmunological framework, linking stress, the cholinergic anti-inflammatory pathway, and direct pro-inflammatory neural circuits to ED pathogenesis. Beyond mechanistic insight, we examine the clinical implications of this connection, discussing the potential of anti-inflammatory therapies and the necessity of integrated management strategies that address both intestinal and sexual health. Our work aims to bridge knowledge gaps and stimulate targeted interventions to improve the quality of life for men living with IBD.

## 1 Introduction

Inflammatory bowel disease (IBD) and erectile dysfunction (ED) are two significant clinical conditions that impose a substantial burden on global health. IBD, encompassing Crohn’s disease and ulcerative colitis, is a chronic inflammatory disorder of the gastrointestinal tract with increasing worldwide incidence (1, 2). ED is defined as the persistent inability to achieve or maintain an erection sufficient for satisfactory sexual intercourse (3). ED is a common male sexual dysfunction, with its prevalence increasing with age and being associated with both physiological and psychological factors (3–5). A growing body of evidence has consistently demonstrated a significantly higher prevalence of ED in male IBD patients compared to the general population (6, 7), suggesting a potential pathophysiological link that extends beyond the mere coincidence of two common conditions. This observed association warrants deeper exploration of the underlying mechanisms and their clinical implications.

Despite the accumulation of epidemiological data, there remains a lack of comprehensive understanding of the mechanistic pathways linking IBD and ED, particularly the interaction between gut-derived inflammation and penile vascular health. Furthermore, clinical guidance for managing ED in this specific patient population remains inadequate (8). Unlike previous reviews primarily focused on epidemiological patterns, psychosocial impacts, and general management principles, this paper aims to provide a unique, mechanism-oriented, comprehensive analysis. We endeavor not only to elucidate the association between the two but also to dissect the precise immunological and vascular pathways along the “gut-penis axis”. A key innovation of this paper is the detailed description of gut-derived signals triggered by barrier dysfunction and dysbiosis. We elucidate how systemic immune cell dysregulation ultimately culminates in cytokine storms that remotely impair penile vascular homeostasis. Concurrently, we critically correlate specific cytokines with molecular events within the corpus cavernosum, thereby refining our understanding of endothelial dysfunction. Moreover, we integrate psychological stress and cholinergic anti-inflammatory pathways into this immunological framework, proposing a coherent psychoneuroimmunological perspective. By delineating the gut-to-penis cascade with heightened mechanistic precision, this review provides a robust theoretical foundation for understanding emerging therapeutic strategies, emphasizing the necessity for targeted interventions beyond conventional ED medications.

## 2 Observational evidence suggests a strong association between IBD and ED

In recent years, an increasing number of observational studies have demonstrated a significant difference in the prevalence of ED between male IBD patients and healthy males. The high prevalence of ED among IBD patients suggests that IBD may play a role in the development, progression, and altered treatment outcomes of male ED.

### 2.1 High prevalence of ED among IBD patients and associated risk factors

The prevalence of ED in male IBD patients is significantly higher than in healthy individuals, with reported rates varying widely from 43% to 92% across studies (9–11). This heterogeneity can be attributed to differences in study populations, settings, and particularly the methods used to define ED. These methods range from self-reported single-item questions to diagnoses based on validated questionnaires, most commonly the International Index of Erectile Function (IIEF-5), with scores of ≤21 or ≤22 (10, 12). A large meta-analysis that accounted for such variability reported a pooled ED prevalence of 27% in IBD patients, firmly establishing IBD as a significant risk factor for ED (13). Similarly, a multicenter prospective cohort study by Gaidos et al. of 171 male veterans with IBD found extremely high rates of sexual dysfunction and ED (12). A cross-sectional study enrolled 208 IBD patients, including 133 with Crohn’s disease and 75 with ulcerative colitis, along with 190 healthy controls (10). Assessment was conducted using the FSFI and IIEF-5 questionnaires. Results showed that the prevalence of ED among male IBD patients was 43.5%, significantly higher than the 12.5% observed in the control group (10). The study revealed a higher incidence of sexual dysfunction among IBD patients, with age, depression, and active perianal disease identified as independent risk factors for ED in males (10). Large-scale systematic reviews and meta-analyses further confirm the high prevalence of ED among IBD patients, establishing it as a significant comorbidity affecting male health in IBD (14, 15). Ageing is a recognized independent risk factor for ED. As men age, sexual function naturally declines, and the presence of IBD further increases the risk of developing ED (9, 12). A large-scale cohort study based on the Taiwan Health Database enrolled 1,845 IBD patients and 7,380 non-IBD control subjects, with a 12-year follow-up period (16). Results revealed that IBD patients exhibited a significantly higher risk of developing ED compared to controls, with a marked age-related correlation. Compared to non-IBD individuals aged ≤49 years, IBD patients aged ≥65 years demonstrated a 3.36-fold increased risk of ED (16). Disease activity in IBD is closely linked to ED, particularly active perianal lesions and active UC (17, 18). Furthermore, IBD-related physical discomfort, altered self-image, and persistent fatigue not only reduce libido and sexual satisfaction but may also impair patients’ ability to maintain satisfying sexual relationships, thereby exacerbating ED (19). Current research evidence indicates that IBD disrupts sexual function through both physiological and psychological pathways, leading to the development of ED.

### 2.2 Observational study on the association between IBD and ED

A growing body of observational evidence demonstrates a significant association between IBD and ED, suggesting that IBD and its related factors contribute substantially to ED pathogenesis. Bidirectional Mendelian randomization analyses suggest a causal effect of IBD on ED risk (20). However, while overall IBD and the CD subtype significantly increase ED risk, some studies have found no significant causal link between UC and ED (21, 22). A clinical controlled study including 358 IBD patients, 110 healthy controls, and 107 IBS patients found a 43% prevalence of ED in male IBD patients, significantly higher than in healthy controls but similar to the IBS group (11). Results showed that the incidence of ED among male IBD patients was 43%, considerably higher than that in healthy controls, with no significant difference compared to the IBS group (11). In males, IBD substantially increases the risk of ED and reduces sexual satisfaction (14). A Danish nationwide cohort study compared 31,498 male IBD patients with 314,980 age-matched controls and found that IBD patients had a higher risk of using ED medications (23). A prospective cross-sectional study involving 85 male patients with IBD revealed an average testosterone concentration of 15.4 nmol/L in this cohort, with 17.6% of participants exhibiting low serum testosterone levels (17). Furthermore, the study identified significantly lower erectile function scores among patients with longer disease duration (17).In summary, evidence from epidemiological, clinical, and genetic studies consistently demonstrates a significant association between IBD (particularly CD) and ED, although the link with UC requires clarification. These findings highlight the need for early ED screening and multidimensional interventions in IBD management.

### 2.3 Critical evaluation of evidence and research limitations

Although observational and genetic studies consistently link IBD and ED, several limitations warrant cautious interpretation. The cross-sectional designs cannot establish causality and are prone to confounding. Large database cohorts offer ample sample sizes but are susceptible to diagnostic inaccuracies and misclassification. Mendelian randomization studies strengthen causal inference and avoid reverse causation. They estimate lifelong genetic risk rather than the effect of modifiable clinical states, and their validity depends on the quality of the genetic instruments. While current evidence strongly implicates IBD as a significant ED risk factor, confirmation from more prospective longitudinal studies is needed. These should enroll IBD patients at baseline and follow them long-term to monitor sexual function changes, while rigorously controlling for the confounding factors mentioned above. Consequently, although the epidemiological link is strong, definitive claims that IBD is a direct and independent cause of ED must be tempered by these potential confounders and methodological constraints. Furthermore, the heterogeneity in ED prevalence across studies may be influenced by several confounding factors. Advanced age is a well-established risk factor for ED and is often correlated with longer IBD duration (9). Disease activity, particularly active perianal disease in CD or active UC, has been consistently associated with worse sexual function (10). Medications commonly used in IBD, such as glucocorticoids, may directly or indirectly affect erectile function through metabolic or mood-related pathways (14, 24, 25). Psychological comorbidities, including depression and anxiety, are highly prevalent in IBD and are independent risk factors for ED (10, 26). Future studies should employ multivariate models to better adjust for these confounders and isolate the independent effect of IBD on ED.

## 3 Potential mechanisms of IBD in the development and progression of ED

Beyond establishing an association, understanding the mechanisms linking IBD and ED is crucial for developing targeted interventions. The interplay involves both immune-inflammatory pathways and psychological abnormalities, which are detailed below.

### 3.1 Immunology and inflammation

The close relationship between IBD and ED is primarily driven by the systemic immune-inflammatory response triggered by IBD. Increasing evidence supports a “gut-penis axis.” Within this framework, local gut immune dysregulation acts as an upstream initiator, driving chronic systemic inflammation that remotely impairs penile neurovascular function. This process involves not only the direct effects of circulating inflammatory factors but also originates from disrupted gut barrier integrity, gut microbiota dysbiosis, and their complex interplay with host immunity. The ensuing cascade from gut to penis is detailed below.

#### 3.1.1 Intestinal barrier dysfunction, microbial translocation, and the initiation of systemic inflammation

**Figure 1:**
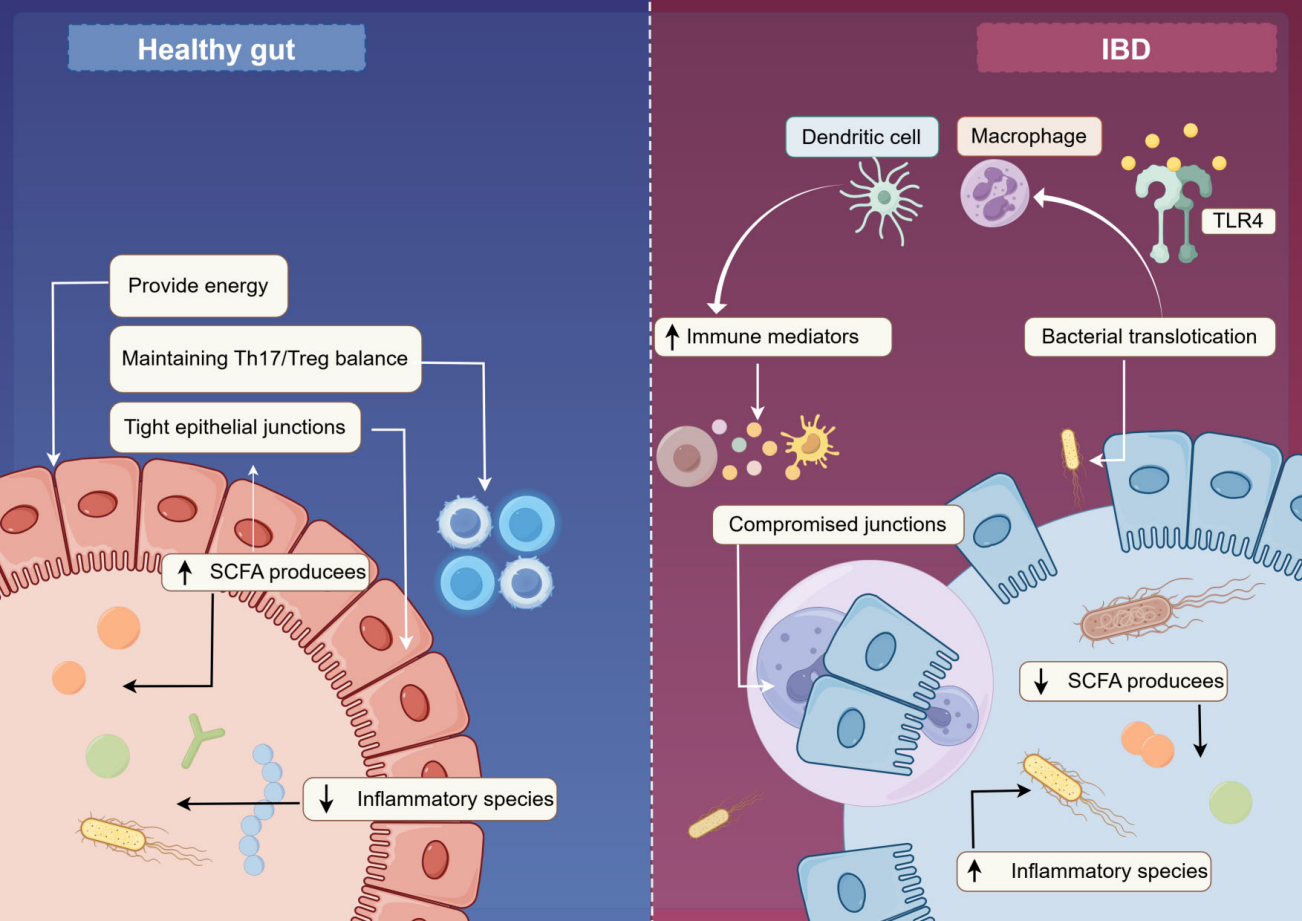
Initiation of systemic inflammation in IBD: from gut barrier dysfunction to immune activation. Schematic diagram illustrating the initiation of systemic inflammation in IBD. The process begins with gut microbiota dysbiosis, characterized by a reduction in beneficial commensal bacteria and a decrease in SCFA production. SCFA deficiency compromises intestinal epithelial barrier integrity by disrupting tight junction proteins. The impaired barrier allows for the translocation of MAMPs, such as LPS, into the lamina propria. MAMPs are recognized by PRRs on innate immune cells, triggering the production of pro-inflammatory cytokines. This cascade initiates a chronic systemic inflammatory state, which serves as the pathological foundation for remote organ dysfunction, including endothelial damage in the penile vasculature.

The core pathophysiological characteristics of IBD are impaired intestinal epithelial barrier function and gut microbiota dysbiosis, which form a vicious cycle (27, 28). A pivotal event in this cycle is a shift in the gut microbiota composition, characterized by a reduction in beneficial commensal bacteria and an expansion of pro-inflammatory species. This dysbiosis directly contributes to the impairment of the epithelial barrier (**Figure 1**). Crucially, reduced beneficial bacteria lower the production of short-chain fatty acids (SCFAs) notably butyrate, acetate, and propionate (29, 30). SCFAs are not only the primary energy source for colonocytes, crucial for maintaining epithelial integrity, but also possess potent anti-inflammatory properties (31). Butyrate strengthens the epithelial barrier by promoting the assembly of tight junction proteins. Furthermore, SCFAs play a fundamental role in immune homeostasis by promoting the differentiation of regulatory T cells (Tregs) and suppressing the generation of pro-inflammatory T helper 17 (Th17) cells, thereby helping to maintain the Th17/Treg balance (30, 32). Consequently, SCFA deficiency compromises both the physical barrier and local immunoregulation. The disrupted barrier leads to increased intestinal permeability, allowing microbial-associated molecular patterns (MAMPs), such as lipopolysaccharide (LPS) from Gram-negative bacteria, to translocate from the intestinal lumen into the lamina propria and systemic circulation (28, 33). Once in the submucosa, MAMPs are recognized by pattern recognition receptors (PRRs), notably Toll-like receptor 4 (TLR4) for LPS, on innate immune cells like macrophages and dendritic cells (33). This activation triggers the production of initial waves of pro-inflammatory cytokines (e.g., TNF-α, IL-1β, IL-6) and drives subsequent adaptive immune responses, culminating in a chronic systemic inflammatory state (29, 34). This gut-originating systemic inflammation is not confined to the digestive tract; evidence suggests that it can directly contribute to and exacerbate pathological changes in the penile vasculature (35).In summary, gut microbiota dysbiosis, through the dual hit of reducing beneficial metabolites and increasing pathogenic stimuli, constitutes the initiating event of IBD-related systemic immune inflammation. This lays the pathological foundation for subsequent systemic endothelial dysfunction, including the vascular lesions in the penile corpora cavernosa.

#### 3.1.2 Systemic immune cell imbalance and cytokine storm driven by gut-derived signals

**Figure 2:**
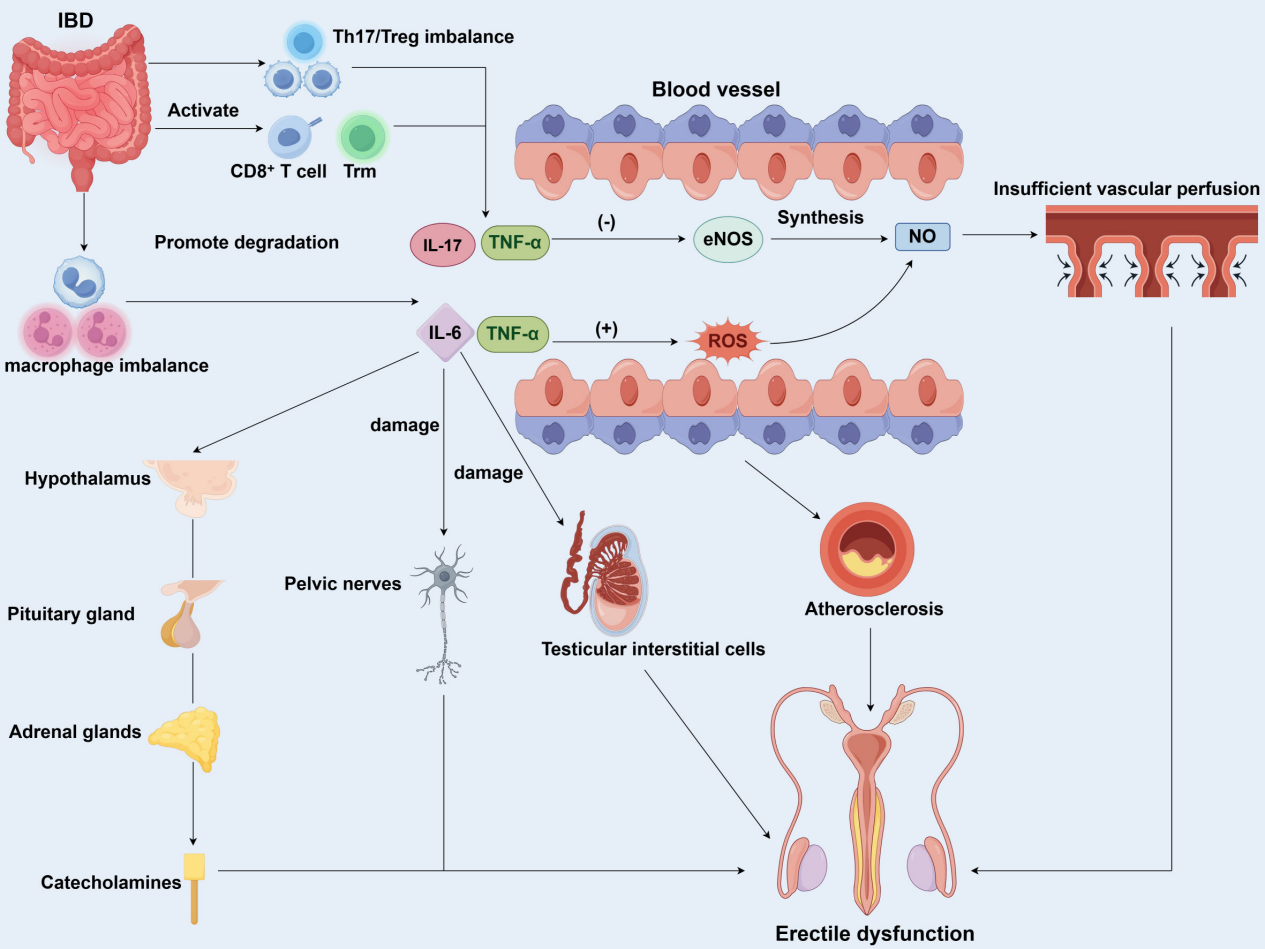
Systemic immune dysregulation and its impact on penile vascular function in IBD-associated ED. Pathophysiological mechanisms by which IBD lead to ED through systemic inflammation. Gut-derived inflammatory signals drive a systemic immune cell imbalance, including Th17/Treg imbalance and M1/M2 macrophage polarization. This results in a pro-inflammatory cytokine storm. These cytokines impair penile vascular endothelial function via two main pathways: (1) Inhibiting eNOS expression and activity, thereby reducing NO synthesis. (2) Inducing ROS that scavenge NO and cause oxidative stress. The combined effect critically compromises cavernous smooth muscle relaxation. Concurrently, chronic inflammation can suppress testosterone synthesis and activate the HPA axis, further exacerbating ED. These mechanisms collectively lead to endothelial dysfunction and inadequate arterial perfusion, culminating in ED.

This systemic immune-inflammatory environment, initiated by gut barrier disruption and dysbiosis, directly impairs penile erectile function (**Figure 2**). In IBD, the intestinal mucosal immune system is abnormally activated, leading to a profound imbalance between pro-inflammatory and anti-inflammatory T cell populations (36–38). There is a marked expansion of pro-inflammatory Th17 cells alongside a relative deficiency in regulatory Tregs, which are responsible for maintaining immune tolerance. This Th17/Treg imbalance leads to excessive production of inflammatory mediators, such as IL-17, which fuels chronic intestinal inflammation and its subsequent spillover into the systemic circulation (34, 38, 39). Furthermore, the role of CD8^+^ T cells is complex and context-dependent, exhibiting both pro-inflammatory and potential regulatory functions influenced by the local microenvironment (40–42). Additionally, the persistence and activation of tissue-resident memory T (Trm) cells within the gut mucosa contribute to the chronicity and relapse of inflammation (43). Concurrently, macrophages are central regulators of immunity and undergo a pathological polarization shift in IBD. There is a predominant polarization towards the pro-inflammatory M1 phenotype, which releases large quantities of TNF-α, IL-1β, and IL-6 (44, 45). In contrast, the anti-inflammatory and tissue-reparative M2 macrophages are relatively suppressed (44–46). This M1/M2 imbalance not only perpetuates gut inflammation but also disrupts systemic immune homeostasis. Critically, M1 macrophages and dysregulated T cells engage in crosstalk via ligand-receptor pairs and cytokine networks, forming a self-amplifying inflammatory loop that sustains high levels of systemic inflammation (35, 45, 47, 48).

This systemic immune-inflammatory environment, triggered by intestinal barrier disruption and microbial imbalance, directly and severely damages penile erectile function (**Table 1**). Elevated pro-inflammatory mediators, such as TNF-α, IL-1β, and IL-6, reach the penile corpus cavernosum through the circulatory system (49). Penile erection is fundamentally a neurovascular event dependent upon endothelium-dependent relaxation. It is well-established that ED shares common pathophysiological pathways with cardiovascular diseases, often serving as a clinical manifestation of systemic endothelial dysfunction (50).These cytokines directly impair the vascular endothelium, which is the cornerstone of erectile function. Specifically, TNF-α and IL-1β have been shown to directly suppress eNOS expression and activity, either by destabilizing its mRNA or inhibiting its phosphorylation, thereby curtailing the synthesis of NO, the principal mediator of penile vasodilation (51–53). Simultaneously, IL-6 and TNF-α are potent inducers of reactive oxygen species (ROS) within the endothelial cells and cavernous smooth muscle (51, 54). This oxidative stress burden is quantifiable by elevated markers such as malondialdehyde (MDA) and reduced antioxidant capacity, which not only accelerate the degradation of NO but also directly damage endothelial cells, creating a vicious cycle of endothelial dysfunction (51, 54). The combined effect of reduced NO synthesis and increased NO scavenging by ROS critically compromises the relaxation capacity of the corpus cavernosum smooth muscle, fundamentally obstructing the hemodynamic process of erection.

**Table 1:** Key cytokine effects penile vascular function in the context of IBD-associated ED.

Cytokine	Molecular/cellular effect on corpus cavernosum	Functional consequence
TNF-α	Suppresses eNOS expression and activity. Induces reactive oxygen species (ROS) production.	Reduces NO synthesis Accelerates NO degradation Promotes endothelial dysfunction
IL-1β	Suppresses eNOS expression and activity.	Reduces NO synthesis Impairs endothelium-dependent relaxation
IL-6	Potent inducer of ROS production.	Accelerates NO degradation Contributes to oxidative stress and endothelial damage
IL-17	Recruits neutrophils and amplifies the local inflammatory response. Synergizes with other cytokines to sustain inflammation	Potentiates vascular inflammation and endothelial activation Indirectly contributes to NO signaling disruption.

eNOS, endothelial nitric oxide synthase; NO, nitric oxide; ROS, reactive oxygen species.

While the systemic inflammation-induced vascular endothelial dysfunction represents the core mechanism of IBD-related ED, it is often compounded by other secondary pathways. The chronic inflammatory state of IBD may also activate the hypothalamic-pituitary-adrenal (HPA) axis, leading to increased catecholamine release and promoting penile vasoconstriction, thereby antagonizing the vasodilation required for erection (17, 19, 55, 56). Additionally, these pro-inflammatory cytokines directly disrupt testosterone production and synthesis in testicular interstitial cells (55, 57). In IBD patients, testosterone concentrations correlate inversely with disease duration, with reduced free testosterone specifically impairing libido and erectile function. Pelvic inflammation or surgery related to IBD may damage the pudendal nerve that innervates the penis, leading to impaired nerve conduction or neuropathy (18, 58). This disrupts the effective transmission of sexual stimulation signals, ultimately causing neurogenic erectile dysfunction (58). Persistent systemic inflammation constitutes an independent risk factor accelerating atherosclerosis. Inflammation damages the vascular endothelium, promoting lipid deposition and plaque formation. When this process affects the internal iliac artery, pudendal artery, and cavernous arteries of the penis, it results in arterial insufficiency (59–61). Therefore, insufficient blood flow required for erection leads to organic ED. In summary, the core mechanism of IBD-related ED involves systemic vascular endothelial damage caused by chronic inflammation, coupled with the combined effects of neuroendocrine dysfunction, leading to the development of ED.

### 3.2 Psychological and mental abnormalities

**Figure 3:**
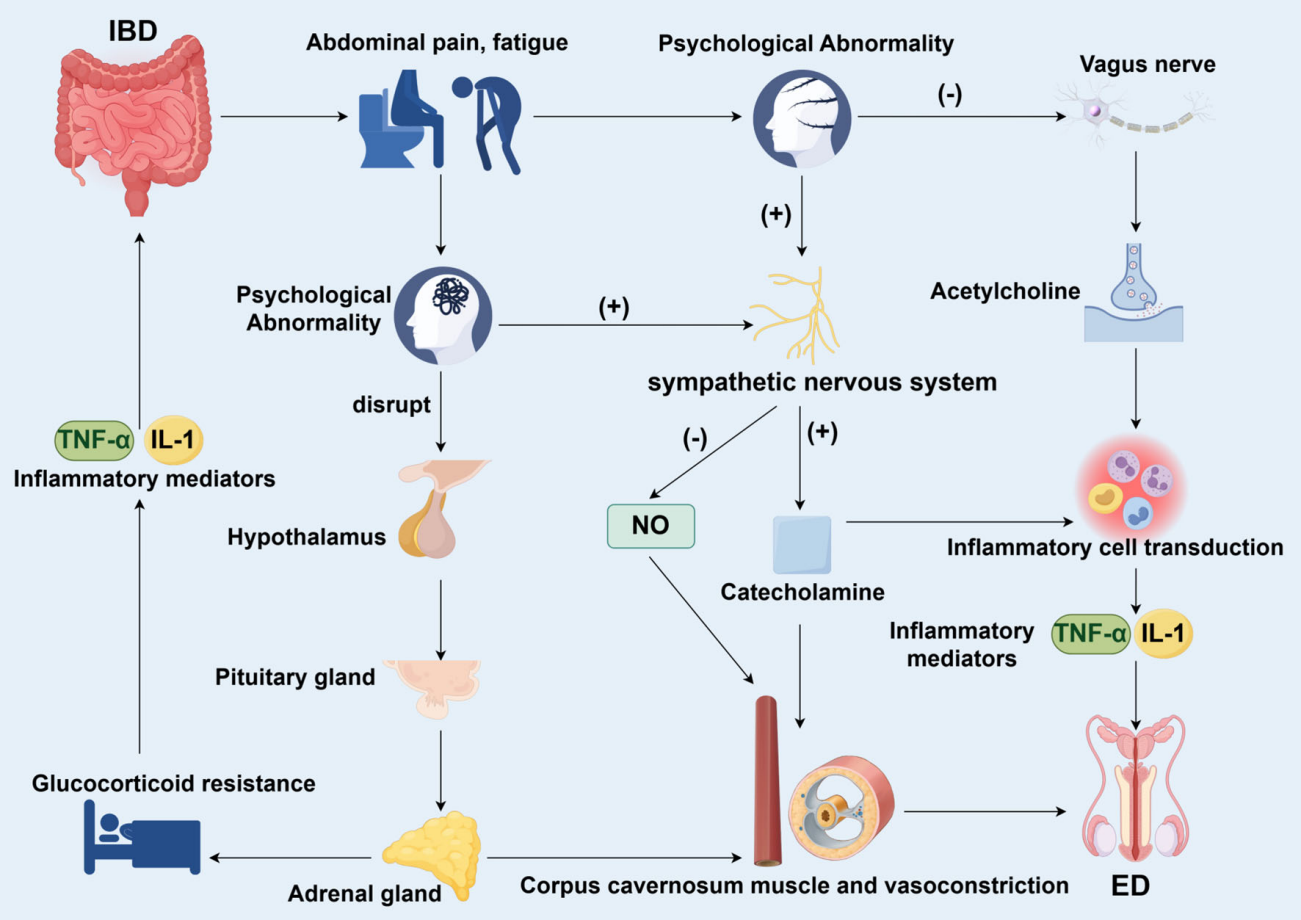
Psychological pathways linking IBD to ED. Schematic illustration of the psychological and mental health mechanisms through which IBD contributes to ED. Chronic gastrointestinal symptoms commonly lead to anxiety and depression in IBD patients. These psychological conditions further diminish libido, disrupt neuroendocrine regulation, and promote sympathetic nervous system hyperactivity, resulting in reduced nitric oxide bioavailability and enhanced penile vasoconstriction. Additionally, emotional distress and diminished quality of life form a self-reinforcing vicious cycle that not only exacerbates intestinal inflammation but also aggravates sexual dysfunction.

The chronic and relapsing inflammation of IBD causes significant psychological distress. Systematic reviews indicate that the pooled prevalence of clinical depression and anxiety in IBD patients reaches 21.6% and 32.1%, respectively, which is substantially higher than in the general population (62, 63). Symptoms such as abdominal pain, diarrhea, and fatigue frequently lead to and exacerbate anxiety and depression, which in turn are strongly implicated in the development of ED in men (**Figure 3**). Critically, this gut-brain communication is bidirectional. Recent research has elucidated that psychological stress can actively drive intestinal inflammation via neural pathways. For instance, Schneider et al. demonstrated that psychological stress activates specific brain nuclei that signal through the enteric nervous system to directly exacerbate intestinal inflammation (64). This establishes a self-reinforcing vicious cycle. Gut inflammation promotes psychological distress, which in turn, via brain-to-gut neural signaling, further fuels the underlying IBD activity, creating a permissive environment for the development and persistence of ED.

Depression is considered one of the most consistent risk factors for SD in IBD patients. Notably, male IBD patients with depressive symptoms exhibit a significantly increased risk of ED, with reported odds ratios (OR) as high as 3.0-6.0 (10, 13). Depression impairs erectile function by reducing libido and disrupting neuroendocrine pathways (26, 65). Psychological stress in IBD patients potently activates the hypothalamic-pituitary-adrenal (HPA) axis and the sympathetic nervous system (SNS). Chronic stress can lead to HPA axis dysregulation, characterized initially by excessive cortisol release and, over time, by glucocorticoid resistance (66–68).This failure to adequately suppress inflammation results in sustained elevation of pro-inflammatory cytokines (66–68). Stress-induced SNS overdrive causes a surge in catecholamines. In the penis, norepinephrine acts on α-adrenergic receptors to promote strong smooth muscle contraction within the corpus cavernosum, directly opposing the vasodilation required for erection and thus facilitating ED (69). Furthermore, catecholamines can bind to adrenergic receptors on immune cells, potentially polarizing them towards a pro-inflammatory phenotype and further fueling the systemic inflammatory fire (70, 71). Beyond the classic stress axes, the vague nerve plays a critical role in the brain-gut connection via the cholinergic anti-inflammatory pathway (72). Vagal efferent fibers release acetylcholine in peripheral tissues, which binds to the α7 nicotinic acetylcholine receptor (α7nAChR) on macrophages and other immune cells, effectively suppressing the production of pro-inflammatory cytokines like TNF-α (73–76). Conversely, recent evidence reveals the existence of direct pro-inflammatory neural circuits. Schneider et al. identified a distinct pathway wherein psychological stress signals from the central amygdala are relayed through the enteric nervous system to directly potentiate intestinal (64). Psychological stress is known to reduce vagal tone, thereby disinhibiting this innate immune response and permitting unchecked inflammation. The impairment of this key anti-inflammatory pathway likely contributes to the persistence of gut inflammation in stressed IBD patients.

And anxiety serves as an independent predictor of ED in patients with IBD, with its severity exhibiting a significant negative correlation with sexual function scores. Studies have shown that the presence of anxiety disorders in IBD males is associated with a more than 3-fold increased risk of ED (OR > 3.0) (10, 13). This anxiety may stem from the uncertainty related to chronic illness, concerns regarding body image, and difficulties within intimate relationships (10, 26, 77). Patients with IBD often experience a comprehensive decline in quality of life, with scores in physical and psychological domains negatively correlated with ED risk (9). Quantitatively, for every 10-point decrease in the mental component summary (MCS) score of the SF-36, the risk of ED increases by approximately 15-20% (9, 78).Impaired emotional quality of life, such as social avoidance and chronic fatigue, directly leads to reduced libido and difficulty maintaining erections (19, 79). Additionally, psychosomatic symptoms like persistent fatigue and sleep disturbances indirectly contribute to ED by diminishing physical stamina and satisfaction in intimate relationships (78–80).IBD indirectly contributes to the development of male ED through psychological abnormalities such as anxiety and depression, as well as reduced quality of life. Psychological factors not only represent independent risk factors for ED but also synergize with the physiological effects of IBD. Therefore, clinical management of IBD patients should integrate psychological assessment and intervention to mitigate the risk of ED.

## 4 Clinical interventions for ED associated with IBD

IBD can impair male sexual function and accelerate ED progression through immune-inflammatory and psychological pathways. For IBD patients, a multifaceted approach is valuable for preventing and treating ED. This includes pharmacologically controlling gut inflammation, implementing mental health and quality-of-life interventions, and employing multidisciplinary collaboration for early ED screening.

### 4.1 Targeted drug therapy

ED is a common complication in IBD patients, significantly impacting quality of life. Controlling IBD-related inflammation is a core strategy for preventing and improving IBD-related ED. Pharmacologic control of IBD alleviates intestinal symptoms and may indirectly benefit sexual function by reducing systemic inflammation, improving endothelial function, and enhancing overall health (23, 81). Anti-TNF-α agents, such as infliximab and adalimumab, form the cornerstone of IBD therapy (82, 83). TNF-α is a key cytokine driving IBD inflammation and directly impairs vascular endothelial function by reducing NO bioavailability (82, 83). Animal studies demonstrate that anti-TNF-α drugs significantly reduce inflammatory marker levels in serum and corpus cavernosum, restore nitric oxide synthase (NOS) expression and testosterone levels to normal, and markedly improve cavernous relaxation function (52, 53). Furthermore, psychological stress represents an independent risk factor for ED in IBD patients. TD Şahin et al. utilized an unpredictable chronic mild stress (UCMS) rat model to demonstrate that chronic psychological stress reduces the immunoreactivity of endothelial nitric oxide synthase (eNOS) and neuronal nitric oxide synthase (nNOS) in the corpus cavernosum, thereby promoting ED development (52). The study further demonstrated that the TNF-α inhibitor infliximab significantly reversed the UCMS-induced decline in eNOS and nNOS expressions (52). JAK inhibitors represent a novel class of drugs clinically used to treat IBD. JAK inhibitors target the Janus kinase-signal transducer and activator of transcription (JAK-STAT) signaling pathway, a crucial cascade initially identified for its role in interferon signaling and now recognized as a master regulator of the expression of multiple pro-inflammatory cytokines (27, 84–86). By inhibiting this pathway, JAK inhibitors have demonstrated efficacy in reducing IBD inflammatory activity in clinical trials, thereby improving mucosal damage and intestinal inflammation (87–90). This effectively controls disease progression and enhances patients’ quality of life (QOL) (80). It is theorized that given the potent anti-inflammatory effects of JAK inhibitors in IBD treatment, they could indirectly exert positive effects on IBD-associated ED. The proposed mechanism would involve reducing systemic and local inflammation levels, thereby mitigating the downstream endothelial dysfunction. However, it is critical to note that current evidence supporting this premise is almost exclusively derived from animal models and theoretical extrapolations of inflammatory pathways (91, 92). Robust clinical data from human interventional trials using sexual function endpoints are currently absent. Therefore, while controlling intestinal inflammation with these agents remains a cornerstone of IBD management, their direct benefit for ED should be considered an important area for future investigation rather than an established conclusion. In addition, clinicians need to be aware of the potential adverse effects of biologics and small-molecule drugs themselves, which may affect sexual function. Although the evidence is inconsistent, some anti-TNF-α drugs and JAK inhibitors have also been reported post-marketing to trigger or worsen mood disorders, which is a well-recognized risk factor for ED. Specific therapies may also indirectly affect libido by interfering with the neuroendocrine axis. Therefore, when developing treatment plans, weighing their benefits in controlling IBD against potential impacts on patients’ sexual health and quality of life is key to achieving individualized precision medicine.

Furthermore, established symptomatic treatments targeting ED directly are indispensable in the comprehensive management of IBD patients. Phosphodiesterase type 5 (PDE5) inhibitors represent first-line pharmacological therapy for ED (23, 93, 94). They act by inhibiting the degradation of cyclic guanosine monophosphate (cGMP), thereby enhancing nitric oxide (NO)-mediated relaxation of corpus cavernosum smooth muscle and effectively improving erectile function (95). Given that one core mechanism of IBD-associated ED is systemic inflammation-induced vascular endothelial dysfunction and impaired NO signaling, PDE5 inhibitors directly target this final common pathological pathway (95). For IBD patients with diagnosed ED, the concomitant use of PDE5 inhibitors alongside active control of intestinal inflammation allows for rapid and effective management of ED symptoms (94, 96). Consequently, IBD-specific therapy and direct ED symptomatic treatment are not mutually exclusive but are synergistic strategies, jointly forming a comprehensive clinical intervention plan for IBD-related ED.

### 4.2 Integrated management: addressing mental health and multidisciplinary collaboration

The management of ED in IBD patients necessitates an integrated approach that addresses both the mind and the body. The prevalence of ED is elevated among patients with IBD and is closely associated with mental health issues, reduced quality of life, and the need for coordinated care. Patients with IBD frequently experience anxiety and depression, psychological states significantly associated with the occurrence of ED. Studies show sexual satisfaction is negatively correlated with depression and anxiety, with anxiety being an independent risk factor for ED (10, 97). ED may further lead to psychological consequences such as low self-esteem and diminished self-image, creating a vicious cycle (98). This complex psychosocial mechanism indicates that the clinical management of ED should extend beyond physiological approaches to incorporate mental health interventions, thereby comprehensively improving patients’ quality of life. Comprehensive interventions, such as IBD-specific cognitive behavioral therapy (CBT), effectively improve sexual dysfunction (99). A randomized controlled trial involving 118 IBD patients revealed that nearly half experienced sexual dysfunction (99). Following an 8-week IBD-specific CBT intervention, not only were depressive symptoms alleviated, but psychological distress and emotional functioning also significantly improved, leading to enhanced sexual performance (99). Therefore, routine screening for psychological disorders and timely provision of psychological support or pharmacological interventions for IBD patients have become integral components of comprehensive ED management (65, 97). ED is also closely associated with multidimensional declines in quality of life among IBD patients, particularly in physical functioning, emotional well-being, and social functioning (9, 80). Overall quality of life improvement has been identified as a key factor in sexual function recovery, particularly as enhancements in emotional and social functioning positively correlate with sexual health. A cross-sectional survey of 202 patients with IBD revealed that male ED is closely associated with depressive symptoms and both gut-related and emotional quality of life (80). Patients with ED demonstrated significantly lower scores in both physical and mental health domains on the SF-36 health-related quality of life scale (9, 12, 80). Overall quality of life improvement has been identified as a key factor in sexual function recovery, particularly as enhancements in emotional and social functioning positively correlate with sexual health.

To operationalize these psychological and quality-of-life interventions effectively and to achieve early screening and prevention of ED, a multidisciplinary team (MDT) approach is essential. In clinical practice, gastroenterologists should collaborate with urologists to systematically assess IBD disease activity and routinely screen for early erectile dysfunction (ED) using standardized tools, such as the IIEF-5, integrating this into standard IBD follow-up protocols (9, 12). Specialized nurses can implement dynamic patient monitoring by incorporating comprehensive health assessments into their daily care routines and facilitating the use of IBD-specific sexual dysfunction scales to more accurately identify disease-related psychosocial factors (19, 80, 100). Laboratory testing systematically monitors serum inflammatory markers and testosterone levels to comprehensively identify high-risk populations for ED (17, 20). Concurrently, psychologists or psychiatrists are crucial for assessing psychological burdens and addressing conditions such as anxiety and depression (10, 101, 102). Cardiovascular specialists may be involved to manage vascular complications exacerbated by chronic inflammation, while urologists or sexual medicine specialists provide targeted treatment for ED. Social workers can further assist in optimizing social support and implementing comprehensive treatment plans (9, 17, 22). Through this interdisciplinary collaboration, controlling disease activity, improving psychological well-being, and managing comorbidities can be synergistically achieved, effectively reducing the risk and impact of ED. Early multidisciplinary intervention not only improves sexual health outcomes but also enhances long-term IBD management by elevating overall quality of life.

## 5 Future research directions

Although an increasing body of evidence has revealed a close association between IBD and male ED, significant gaps remain in current understanding. The core limitation is that evidence supporting improvements in ED with anti-TNF-α and JAK inhibitors primarily comes from animal studies and theoretical extrapolations. At the same time, data on sexual function endpoints from human clinical trials remain scarce. Therefore, future research should focus on multiple levels. At the mechanistic level, it is necessary to employ preclinical models to characterize the penile endothelial transcriptome and proteome, identifying dysregulated pathways beyond broad inflammatory responses. Concurrently, single-cell RNA sequencing of human corpus cavernosum tissues, when available, should be utilized to map cell-type-specific responses to systemic inflammation, identifying novel therapeutic targets. And utilize large-scale genetic and prospective clinical studies to clarify subtype differences in CD and UC in their association with ED. At the therapeutic translation level, specialized clinical trials incorporating sexual function endpoints are urgently needed to verify the potential value of emerging therapies such as JAK inhibitors; currently, the evidence for these agents improving ED is preclinical and requires human validation. More importantly, randomized controlled trials should systematically assess the synergistic effects and safety of combined PDE5 inhibitors and anti-inflammatory therapies, while also paying attention to the potential impacts of various IBD drugs themselves on the gonadal axis and mood, to comprehensively weigh benefits against risks. At the clinical management level, integrated management models that combine psychological interventions with multidisciplinary team (MDT) collaboration need to be developed and validated. Ultimately, by identifying reliable biomarkers to enable early screening and personalized intervention, it will be possible to fundamentally improve sexual health and overall quality of life in male patients with IBD.

## 6 Conclusion

This review summarizes the significant epidemiological association between IBD and ED, the multi-level pathophysiological mechanisms involved, and potential clinical intervention strategies. Substantial evidence indicates a markedly elevated prevalence of ED among IBD patients, with mechanisms involving multiple pathways, including chronic inflammation-mediated vascular endothelial dysfunction, neuroendocrine dysregulation, oxidative stress, and psychosocial factors. In clinical management, controlling intestinal inflammatory activity by employing targeted therapies such as anti-TNF-α and JAK inhibitors, combined with psychological interventions and MDT approaches, is of significant importance for preventing and improving IBD-related ED. Future research should further clarify the causal relationship between different IBD subtypes and ED, delve deeper into molecular mechanisms, conduct targeted clinical trials, and establish early screening and individualized intervention strategies to comprehensively enhance sexual health and quality of life for male IBD patients.
